# Exploring the MicroRNA Landscape in Cardiac Amyloidosis: Molecular Insights and Clinical Applications

**DOI:** 10.3390/genes17030356

**Published:** 2026-03-23

**Authors:** Joanna E. Kontaraki, Anthoula Plevritaki, Aleksi Sallo, Konstantinos Fragkiadakis, Eleutherios Kallergis, Evangelos Zacharis, John Kopidakis, Emmanouil Kampanieris, Sophia Achladianaki, Vasiliki Papakosta, Emmanouil Simantirakis, Maria E. Marketou

**Affiliations:** 1 Molecular Cardiology Laboratory, School of Medicine, University of Crete, 70013 Heraklion, Crete, Greece; salloalex309@gmail.com (A.S.); maryemarke@uoc.gr (M.E.M.); 2 Cardiology Department, Heraklion University General Hospital, 71500 Heraklion, Crete, Greece; anthiplevritaki@gmail.com (A.P.); fragkiadakisk@hotmail.com (K.F.); ekallergis@med.uoc.gr (E.K.); ezacharis@yahoo.gr (E.Z.); kopidakisg@gmail.com (J.K.); manoskampanieris.94@gmail.com (E.K.); sofiaeirini22@yahoo.gr (S.A.); vasopapacosta@gmail.com (V.P.); simantir@uoc.gr (E.S.)

**Keywords:** cardiac amyloidosis, transthyretin, immunoglobulin light chains, microRNAs

## Abstract

Background: Cardiac amyloidosis (CA) is an increasingly recognized cause of heart failure with preserved ejection fraction, resulting from myocardial deposition of misfolded amyloid fibrils derived predominantly from transthyretin (ATTR wild-type [ATTRwt] or variant [ATTRv]) or immunoglobulin light chains (AL). Despite advances in noninvasive imaging and disease-modifying therapies, delayed diagnosis remains common, and clinically actionable molecular biomarkers for early detection, phenotypic discrimination, and therapeutic monitoring are limited. MicroRNAs (miRNAs), small noncoding regulators of post-transcriptional gene expression, have emerged as key modulators of cardiovascular remodeling and systemic amyloid biology. Methods: We performed a comprehensive review of experimental, translational, and clinical studies to evaluate the role of miRNAs in transthyretin and light-chain cardiac amyloidosis, incorporating data from myocardial tissue analyses, circulating miRNA profiling, and mechanistic studies in cellular and animal models. Results: Dysregulated miRNA networks contribute to amyloid-induced cardiac injury by modulating mitochondrial energetics, oxidative stress, inflammation, fibrosis, proteostasis, and neurocardiac signaling. Specific miRNAs, including members of the miR-21, miR-29, and miR-30 families, as well as miR-150-5p and miR-339, have been associated with amyloid burden, adverse myocardial remodeling, plasma cell biology, and disease severity. Distinct circulating and tissue miRNA signatures differentiate transthyretin from light-chain cardiac amyloidosis and correlate with functional status, heart failure biomarkers, and clinical outcomes. Conclusions: MiRNAs represent promising diagnostic and prognostic biomarkers in cardiac amyloidosis and offer mechanistic insights into disease pathogenesis. Integration of miRNA profiling with multimodality imaging and emerging RNA-based therapeutics may enable earlier diagnosis and support precision management of amyloid-related heart failure.

## 1. Introduction

Cardiac amyloidosis (CA) is an infiltrative cardiomyopathy caused by extracellular deposition of misfolded amyloid fibrils within the myocardium, leading to progressive ventricular stiffening, diastolic dysfunction, and ultimately heart failure [1,2,3,4]. Once considered a rare disease, CA is now increasingly recognized as an underdiagnosed cause of heart failure, particularly heart failure with preserved ejection fraction (HFpEF), owing to advances in imaging techniques and heightened clinical awareness [5,6,7]. The two most common etiologies are transthyretin amyloidosis (ATTR), resulting from deposition of wild-type or mutant transthyretin, and immunoglobulin light-chain amyloidosis (AL), caused by monoclonal plasma cell-derived light chains. AL amyloidosis results from a plasma cell dyscrasia and is characterized by rapid disease progression and high early mortality if untreated. ATTR amyloidosis, caused by misfolded transthyretin protein, occurs either as a hereditary disease due to pathogenic variants or as a wild-type form associated with aging [8,9]. Despite important differences in pathogenesis, both forms ultimately converge on a shared phenotype of restrictive cardiomyopathy and progressive myocardial dysfunction. The clinical phenotype of CA overlaps substantially with other forms of hypertrophic or restrictive cardiomyopathy, frequently resulting in delayed diagnosis and advanced disease at presentation. Although noninvasive imaging modalities—including bone-avid scintigraphy, cardiac magnetic resonance, and echocardiographic strain imaging—have transformed diagnostic pathways, they primarily detect established myocardial involvement. Thus, limitations persist in early disease detection, disease monitoring, and risk stratification [10,11]. Similarly, contemporary therapies such as transthyretin stabilizers, gene-silencing agents, and plasma cell-directed regimens improve outcomes but are most effective when initiated early. Accordingly, there is a critical unmet need for molecular biomarkers that reflect early disease biology, enable etiologic differentiation, and provide insight into disease activity and therapeutic response.

MicroRNAs (miRNAs) are short, noncoding RNA molecules that regulate gene expression through post-transcriptional repression of target messenger RNAs. By orchestrating complex transcriptional networks, miRNAs play essential roles in cardiovascular development, myocardial remodeling, mitochondrial homeostasis, inflammation, and fibrosis. Dysregulation of miRNA expression has been implicated in a wide spectrum of cardiovascular diseases, including heart failure, hypertrophic cardiomyopathy, and ischemic heart disease. Importantly, miRNAs are detectable in both cardiac tissue and circulation, conferring potential utility as minimally invasive biomarkers [12,13,14].

In recent years, accumulating evidence has linked specific miRNA signatures to the pathophysiology of both ATTR and AL cardiac amyloidosis. These miRNAs modulate key disease processes, including amyloid precursor protein handling, mitochondrial dysfunction, oxidative stress, extracellular matrix remodeling, microvascular dysfunction, and neurocardiac cross-talk. Moreover, distinct miRNA expression profiles appear to differentiate ATTR from AL cardiomyopathy and correlate with disease severity, functional impairment, and prognosis.

In this review, we provide a comprehensive synthesis of current evidence on the mechanistic roles, diagnostic potential, and therapeutic implications of miRNAs in cardiac amyloidosis. Emphasis is placed on pathways central to heart failure pathophysiology, including mitochondrial energetics, myocardial fibrosis, inflammation, and microvascular dysfunction, as well as emerging RNA-based therapeutic strategies. By integrating molecular insights with clinical relevance, we highlight the potential of miRNA profiling to complement multimodality imaging and advance precision medicine in amyloid-related heart failure.

## 2. Pathophysiology of Cardiac Amyloidosis

### 2.1. General Mechanisms of Amyloid-Related Cardiac Dysfunction

CA is characterized by the extracellular deposition of insoluble amyloid fibrils with a β-pleated sheet conformation within the myocardial interstitium. Progressive fibril accumulation disrupts myocardial architecture, increases ventricular stiffness, impairs diastolic filling, alters electrical conduction, and ultimately culminates in restrictive cardiomyopathy [1,2,3]. Amyloid fibrillogenesis is initiated by protein destabilization resulting from inherited mutations in transthyretin (ATTRv), age-related conformational instability of wild-type transthyretin (ATTRwt), or the overproduction of misfolded κ or λ immunoglobulin light chains in plasma cell dyscrasias (AL amyloidosis) [15,16,17,18,19,20]. These unstable monomers assemble into soluble oligomers and protofibrils before forming mature amyloid fibrils. Increasing evidence indicates that soluble oligomeric species, rather than deposited fibrils, exert the greatest cardiotoxic effects by inducing endothelial dysfunction, oxidative stress, intracellular calcium dysregulation, and cardiomyocyte apoptosis [4,5,6,7,21,22]. The pathological hallmark of CA is diffuse amyloid infiltration of the myocardial extracellular matrix, leading to increased ventricular wall thickness, reduced compliance, and impaired ventricular relaxation. Unlike hypertrophic cardiomyopathy, myocardial thickening in CA reflects infiltrative expansion rather than myocyte hypertrophy, resulting in a restrictive physiology that progressively worsens over time [8,23]. This structural remodeling profoundly compromises cardiac filling and output, particularly during exertion.

In AL amyloidosis, circulating monoclonal light chains exert direct cardiotoxic effects independent of fibril deposition, promoting oxidative stress, mitochondrial dysfunction, and cardiomyocyte apoptosis. This dual pathogenic mechanism—amyloid infiltration combined with proteotoxic injury—accounts for the rapid clinical deterioration and poor prognosis commonly observed in AL cardiac amyloidosis [4,24]. In contrast, ATTR amyloidosis typically follows a more indolent course, with chronic transthyretin-derived fibril accumulation leading to progressive myocardial stiffening and fibrosis.

Amyloid deposition also disrupts intracellular calcium handling and electromechanical coupling, predisposing patients to atrial and ventricular arrhythmias as well as conduction system disease [25,26,27,28,29]. Concomitant microvascular dysfunction and endothelial impairment further exacerbate myocardial ischemia and accelerate disease progression [30,31]. Fibrotic remodeling represents a key downstream consequence of amyloid infiltration and correlates closely with disease severity and prognosis. Cardiac magnetic resonance imaging has demonstrated that the extent of late gadolinium enhancement reflects both amyloid burden and interstitial fibrosis, underscoring the contribution of secondary remodeling processes to adverse clinical outcomes [32].

### 2.2. Transthyretin Cardiac Amyloidosis

Transthyretin (ATTR) amyloidosis results from destabilization of the transthyretin tetramer, leading to dissociation into misfolded monomers that aggregate into insoluble amyloid fibrils. Wild-type ATTR amyloidosis is increasingly recognized as an age-related systemic disorder, predominantly affecting the heart and frequently associated with extracardiac manifestations such as carpal tunnel syndrome, lumbar spinal stenosis, and spontaneous biceps tendon rupture [33,34,35,36]. Cardiac involvement typically evolves gradually and is characterized by progressive myocardial infiltration and restrictive physiology [37,38]. Hereditary ATTR amyloidosis arises from pathogenic mutations in the transthyretin gene that promote tetramer instability and influence fibril formation and tissue tropism. Depending on the specific variant, clinical presentation may range from predominantly cardiac to mixed cardiac–neurologic phenotypes [39]. Key pathophysiologic features of ATTR amyloidosis include progressive extracellular deposition of amyloid fibrils within the myocardial interstitium, accompanied by chronic low-grade inflammation and activation of hypoxia-responsive signaling pathways. These processes contribute to microvascular dysfunction with impaired myocardial perfusion, mitochondrial energetic failure, and sustained metabolic stress. In ATTR cardiomyopathy, mitochondrial dysfunction is increasingly recognized as a central pathophysiologic mechanism contributing to impaired myocardial bioenergetics and oxidative stress. Studies using positron emission tomography have demonstrated disturbed myocardial oxidative metabolism and reduced mitochondrial oxidative function in wild-type ATTR-CM, indicating defective mitochondrial energetics [40]. Experimental data also show that ATTR-associated pathological processes are linked with damaged mitochondrial ultrastructure, decreased mitochondrial number, elevated reactive oxygen species, and impaired respiration capacity, consistent with mitochondrial dysfunction in disease progression [41].

Proteomic and transcriptomic studies demonstrate disrupted oxidative phosphorylation, altered mitochondrial dynamics, and impaired bioenergetic reserve, contributing to exercise intolerance and heart failure progression in transthyretin amyloid cardiomyopathy. In patients with wild-type ATTR-CM, myocardial mitochondrial oxidative phosphorylation capacity is reduced and correlates with impaired exercise hemodynamics, indicating defective energy metabolism early in disease progression [40]. Recent proteomic profiling of ATTR myocardium reveals downregulated metabolic pathways consistent with disrupted cardiomyocyte energetics, and omics analyses have explicitly identified mitochondrial oxidative phosphorylation dysfunction and altered mitochondrial regulatory pathways as key contributors to myocardial dysfunction and clinical exercise intolerance in ATTR-CM [42]. Importantly, in contrast to AL amyloidosis, ATTR cardiomyopathy is characterized by a relative absence of direct cardiomyocyte toxicity. Instead, myocardial dysfunction arises predominantly from infiltrative burden, metabolic derangements, and microvascular impairment rather than acute cytotoxic injury [37,43,44].

### 2.3. Immunoglobulin Light-Chain Cardiac Amyloidosis

Immunoglobulin light-chain (AL) amyloidosis is a rapidly progressive multisystem disease associated with a plasma cell dyscrasia and remains the most aggressive form of cardiac amyloidosis. In contrast to ATTR, myocardial dysfunction in AL amyloidosis is driven not only by amyloid fibril deposition but also by the direct cardiotoxic effects of circulating monoclonal light chains. Experimental and clinical studies have demonstrated that amyloidogenic light chains induce oxidative stress, mitochondrial injury, and cardiomyocyte apoptosis, leading to profound diastolic dysfunction and early hemodynamic compromise. Physiological levels of human amyloidogenic light chains increase intracellular ROS and impair calcium handling and contractile/relaxation function in isolated cardiomyocytes, independent of fibril deposition [4]. Patient-derived light chains also generate ROS, leading to significant mitochondrial ultrastructural damage in vivo, mimicking cardiac injury [45]. Proteomic analyses show LC-induced apoptosis and oxidative stress in cardiac cells, and murine LC injection models demonstrate cardiomyocyte apoptosis with reduced cardiac performance even without amyloid deposits [21]. Mechanistically, LC activation of p38α MAPK underlies oxidative stress, contractile dysfunction, and apoptosis in cardiomyocytes [46]. Microvascular dysfunction, endothelial impairment, and nitric oxide depletion further exacerbate myocardial injury. Without prompt treatment, cardiac AL amyloidosis frequently progresses rapidly to advanced heart failure and cardiogenic shock. Compared with ATTR amyloidosis, AL amyloidosis is associated with substantially higher early mortality, often occurring within months of diagnosis if left untreated. Key pathophysiologic features of immunoglobulin light-chain (AL) amyloidosis include severe restrictive diastolic dysfunction driven by myocardial infiltration, coupled with direct light chain-mediated cardiotoxicity that induces oxidative stress, mitochondrial injury, and cardiomyocyte apoptosis. These processes are accompanied by pronounced microvascular dysfunction and endothelial damage, contributing to impaired myocardial perfusion. In the absence of prompt therapy, AL cardiac amyloidosis is characterized by rapid clinical progression and high early mortality [5].

## 3. Molecular Basis of Amyloid-Induced Cardiac Injury

Cardiac involvement in amyloidosis arises from a multifactorial pathogenic cascade encompassing extracellular amyloid fibril deposition, intracellular stress responses, mitochondrial dysfunction, immune activation, microvascular injury, and progressive disruption of myocardial architecture. Although ATTR and AL amyloidosis converge clinically on a restrictive cardiomyopathy phenotype, their molecular drivers differ substantially. ATTR cardiomyopathy is largely characterized by progressive extracellular infiltration and chronic mechanical and metabolic stress, whereas AL cardiac amyloidosis (AL-CA) is dominated by direct cardiotoxic effects of circulating light chains. These mechanistic distinctions are critical for interpreting disease-specific microRNA dysregulation patterns.

### 3.1. Extracellular Matrix Remodeling and Fibrosis

**Figure 1:**
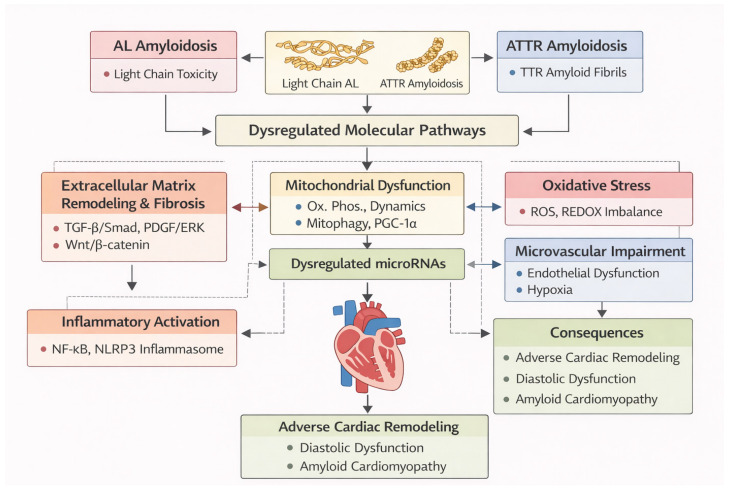
Molecular basis of amyloid-induced cardiac injury. Schematic representation of amyloid-induced molecular injury in cardiac amyloidosis. Deposition of immunoglobulin light-chain (AL) or transthyretin-derived (ATTR) amyloid fibrils disrupts myocardial homeostasis through activation of fibrotic signaling, mitochondrial dysfunction, oxidative stress, microvascular impairment, and inflammatory pathways. These processes are modulated by dysregulated microRNA networks, which contribute to adverse cardiac remodeling, diastolic dysfunction, and progression of amyloid cardiomyopathy. Abbreviations: ROS, reactive oxygen species; REDOX, reduction oxidation; TGF-β, Transforming growth factor beta; Smad, Small mothers against decapentaplegic (SMAD family proteins); PDGF, Platelet-derived growth factor; ERK, Extracellular signal-regulated kinase; PGC-1α, Peroxisome proliferator-activated receptor gamma coactivator 1-alpha.

Amyloid deposition activates resident cardiac fibroblasts, leading to excessive extracellular matrix (ECM) expansion and diffuse interstitial fibrosis. Central signaling pathways include transforming growth factor-β (TGF-β)/Smad-mediated induction of collagen types I and III, platelet-derived growth factor (PDGF)/extracellular signal-regulated kinase (ERK)-driven fibroblast proliferation, mitogen-activated protein kinase (MAPK) signaling, and Wnt/β-catenin activation, which collectively promote extracellular matrix accumulation while suppressing matrix degradation. TGF-β1 activates Smad3 to drive transcription of profibrotic genes including collagen I and III in cardiac fibroblasts and post-injury myocardium [47]. PDGFR-β engagement stimulates ERK signaling to enhance fibroblast proliferation and ECM gene expression [48]. Non-canonical TGF-β signaling activates MAPK pathways—namely ERK, c-Jun N-terminal kinase (JNK), and p38 MAPK—thereby reinforcing fibroblast activation and promoting extracellular matrix gene expression [49]. Canonical Wnt/β-catenin signaling promotes myofibroblast differentiation and potentiates TGF-β-driven ECM production in cardiac fibroblasts [50] (Figure 1).

MiRNAs act as critical post-transcriptional regulators of these profibrotic pathways and play a central role in shaping the fibrotic response in amyloid cardiomyopathy. Experimental studies demonstrate that miRNAs such as miR-29b directly repress TGF-β/Smad3-mediated collagen gene expression in cardiac fibrosis models, while miR-101a and miR-331 modulate TGF-β receptor signaling and downstream Smad activation, thereby reducing fibroblast activation and ECM production [51,52,53].

### 3.2. Mitochondrial Dysfunction

Mitochondrial dysfunction has emerged as a core pathogenic mechanism in cardiac amyloidosis, particularly in AL disease but also in ATTR cardiomyopathy. Proteomic and transcriptomic analyses of amyloid-infiltrated myocardium demonstrate widespread disruption of mitochondrial energetics, ultrastructure, and quality-control systems in both subtypes [4,54]. Experimental and translational studies reveal impaired oxidative phosphorylation, reduced electron transport chain efficiency, and diminished ATP generation [54]. Alterations in mitochondrial dynamics, characterized by an imbalance between fission and fusion processes, further contribute to mitochondrial instability and cardiomyocyte dysfunction [55,56]. Defective mitochondrial quality control, including suppression of PTEN-induced kinase 1 (PINK1)/Parkin-dependent mitophagy, results in accumulation of damaged mitochondria and heightened cellular stress [57]. Excess reactive oxygen species production and metabolic inflexibility amplify inflammatory and pro-fibrotic signaling. In parallel, downregulation of key regulators of mitochondrial biogenesis—peroxisome proliferator-activated receptor-γ coactivator-1α (PGC-1α), nuclear respiratory factor-1 (NRF1), and mitochondrial transcription factor A (TFAM)—limits adaptive energetic responses in amyloid cardiomyopathy [54,58]. MiRNAs targeting these mitochondrial regulatory networks, including those modulating PGC-1α, dynamin-related protein-1 (DRP1), BCL2 Interacting Protein 3 (BNIP3), and the PINK1/Parkin axis, provide a molecular link between proteotoxic stress, impaired mitochondrial homeostasis, and disease progression [43,59] (Figure 1).

### 3.3. Microvascular Rarefaction and Hypoxia

Both AL and ATTR amyloidosis impair endothelial function, reduce nitric oxide bioavailability, and promote microvascular rarefaction, thereby exacerbating myocardial hypoxia. Emerging evidence suggests that age-related hypovascularity synergizes with amyloid-induced endothelial dysfunction to activate hypoxia-inducible factor-1α (HIF-1α) signaling and downstream inflammatory cascades, further aggravating myocardial injury [43,44] (Figure 1).

## 4. MicroRNA Biology and Relevance to Amyloidosis

MicroRNAs are short (18–24 nucleotides), noncoding RNA molecules that regulate post-transcriptional gene expression through sequence-specific binding to complementary regions within target mRNAs, resulting in translational repression or mRNA degradation [13,60]. In the cardiovascular system, miRNAs orchestrate complex gene regulatory networks involved in cardiomyocyte survival, fibrosis, inflammation, and vascular homeostasis, processes that are centrally implicated in amyloid cardiomyopathy. Circulating miRNA profiling studies in patients with cardiac amyloidosis have identified distinct circulating miRNA signatures: miR-339-3p was significantly upregulated in senile cardiac amyloidosis compared with healthy controls, indicating disease-specific expression patterns in the bloodstream [61]. In transthyretin variant (ATTRv) amyloidosis patients, a set of differentially expressed circulating miRNAs, including miR-150-5p, were identified and validated, linking circulating miRNA dysregulation with symptomatic cardiomyopathy [62].

Proteostasis—the balance between protein synthesis, folding, and degradation—is a defining feature of amyloid diseases. Although direct evidence in cardiac amyloidosis remains limited, miRNAs are known regulators of ubiquitin–proteasome activity, autophagy, and endoplasmic reticulum stress responses in cardiovascular disease models [13,63]. In addition, miRNAs modulate ECM turnover, mitochondrial function, immune activation, endothelial integrity, and cardiomyocyte hypertrophy, all of which contribute to amyloid-mediated myocardial dysfunction [12,64]. Despite the relatively early stage of investigation, circulating miRNA profiling studies in cardiac amyloidosis have identified reproducible disease-associated signatures. Importantly, certain circulating miRNAs are detectable in peripheral blood and show disease-related alterations, supporting their potential role as minimally invasive biomarkers in amyloid cardiomyopathy [61].

### 4.1. Differential miRNA Expression in AL vs. ATTR Cardiomyopathy

The first systematic blood-based miRNA profiling study in cardiac amyloidosis demonstrated distinct expression patterns compared with healthy controls, with miR-339-3p emerging as a significantly upregulated candidate with discriminatory potential, highlighting the feasibility of miRNA-based diagnostics in this population [61]. While direct comparative studies between AL and ATTR cardiomyopathy remain limited, fundamental pathophysiological differences between the two entities suggest divergent miRNA involvement.

**Figure 2:**
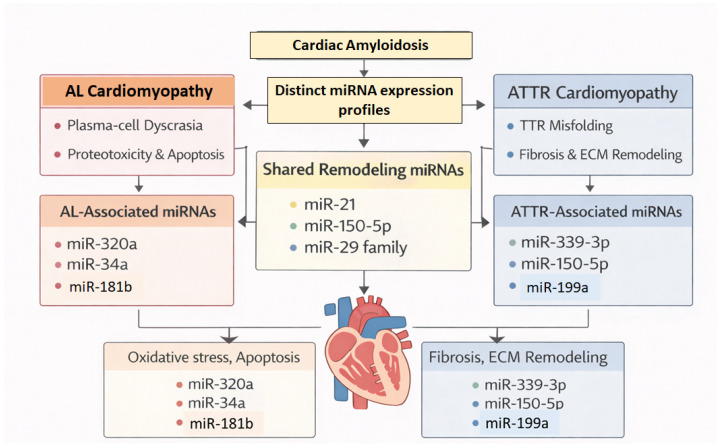
Differential microRNA expression in AL versus ATTR cardiomyopathy. Overview of disease-specific microRNA (miRNA) signatures in cardiac amyloidosis. Light-chain (AL) cardiomyopathy is associated with miRNAs linked to oxidative stress, apoptosis, and cardiotoxicity, whereas transthyretin (ATTRwt and ATTRv) cardiomyopathy is characterized by miRNAs regulating fibroblast activation and extracellular matrix (ECM) remodeling. Distinct circulating and tissue miRNA profiles reflect these divergent pathogenic mechanisms and highlight the potential of miRNAs as minimally invasive biomarkers for disease classification and monitoring.

AL cardiomyopathy is driven by direct light-chain-mediated cardiotoxicity, leading to oxidative stress, apoptosis, and rapid myocardial dysfunction. In contrast, ATTR cardiomyopathy evolves through progressive extracellular amyloid deposition, chronic myocardial stiffening, and fibrotic remodeling [65,66]. Extrapolating from broader cardiovascular literature, AL disease is therefore more likely to involve apoptosis- and oxidative stress-related miRNAs, whereas ATTR cardiomyopathy may preferentially engage miRNAs regulating fibroblast activation and ECM remodeling [13,64] (Figure 2).

### 4.2. Pro-Fibrotic miRNAs in Amyloid Cardiomyopathy

**Figure 3:**
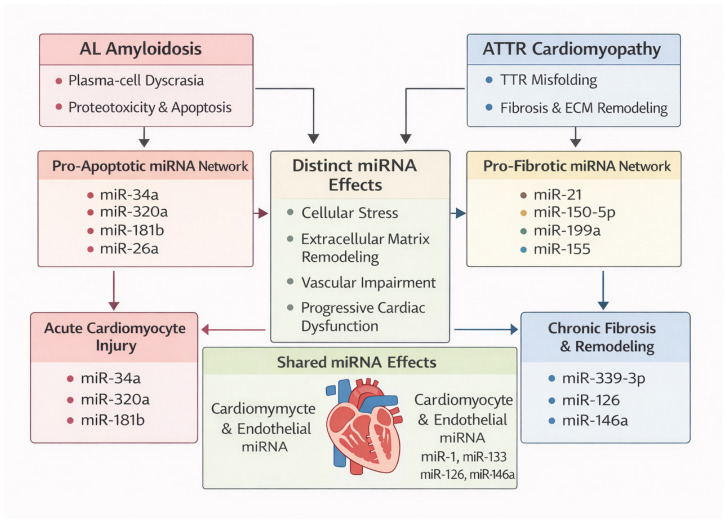
Mechanistic roles of disease-associated microRNAs in amyloid cardiomyopathy. Schematic summary of key microRNA (miRNA) networks implicated in myocardial injury and remodeling in cardiac amyloidosis. Pro-fibrotic miRNAs, including miR-21, miR-199a, and miR-155, promote fibroblast activation and inflammatory–fibrotic signaling, contributing to extracellular matrix expansion, particularly in transthyretin (ATTR) cardiomyopathy. In contrast, anti-fibrotic and ECM-regulatory miRNAs, most notably the miR-29 family, repress collagen expression and modulate matrix remodeling. In light-chain (AL) amyloidosis, miRNAs associated with cardiomyocyte stress and apoptosis, including miR-34a, miR-320a, and miR-181b, reflect direct light-chain-mediated cardiotoxicity and mitochondrial dysfunction. Cardiomyocyte- and endothelial-enriched miRNAs, such as miR-1, miR-133, miR-126, and miR-146a, regulate electrical stability, survival pathways, angiogenesis, and inflammatory signaling. Together, these miRNA networks link amyloid burden to fibrosis, cellular stress, vascular dysfunction, and progressive cardiac impairment.

Myocardial fibrosis is a defining feature of ATTR cardiomyopathy and a major determinant of diastolic dysfunction. Among pro-fibrotic miRNAs, miR-21 is a well-established regulator of cardiac fibroblast survival and TGF-β signaling in multiple cardiomyopathies [67]. Although direct ATTR-specific mechanistic studies are lacking, the pronounced fibrotic phenotype of ATTR cardiomyopathy supports a contributory role for miR-21-mediated pathways. Additional miRNAs with pro-fibrotic and pro-inflammatory effects, including miR-199a and miR-155, promote fibroblast proliferation and inflammatory–fibrotic signaling in experimental and clinical cardiovascular disease, providing further mechanistic plausibility for their involvement in amyloid-associated myocardial remodeling [68,69] (Figure 3).

### 4.3. Anti-Fibrotic and ECM-Regulatory miRNAs

The miR-29 family plays a central role in ECM homeostasis by repressing the expression of collagen types I and III and fibrillin [70]. Downregulation of miR-29 has been consistently linked to pathological myocardial fibrosis across diverse cardiac disease states. In amyloid cardiomyopathy, suppression of miR-29-mediated ECM regulation represents a plausible molecular mechanism contributing to early matrix expansion and increased myocardial stiffness, even before overt systolic dysfunction becomes apparent [66,70] (Figure 3).

### 4.4. Apoptosis and Cardiomyocyte Stress miRNAs

AL amyloidosis is uniquely characterized by direct light-chain-mediated cardiotoxicity, leading to cardiomyocyte apoptosis and biomarker release independent of amyloid burden. MiRNAs such as miR-34a, miR-320a, and miR-181b have been implicated in cardiomyocyte apoptosis, oxidative stress, and mitochondrial dysfunction in cardiovascular disease and heart failure cohorts [71,72,73]. Although not yet validated specifically in AL amyloidosis, these miRNAs represent biologically plausible mediators of light-chain-induced myocardial injury.

In contrast, ATTR cardiomyopathy is associated with less prominent apoptotic signaling and is dominated by chronic metabolic stress and low-grade inflammation. In this context, miR-146a—a key regulator of innate immune and NF-κB signaling—has been linked to inflammatory and metabolic stress responses and may contribute to ATTR-related myocardial dysfunction [74].

In addition to apoptosis, amyloid deposition induces direct cardiomyocyte toxicity, resulting in cellular stress, impaired calcium handling, and eventual cell death. Cardiomyocyte-enriched miRNAs such as miR-133 and miR-1 play key roles in regulating hypertrophy, electrical stability, and survival pathways [64]. Dysregulation of these miRNAs has been associated with myocardial injury and adverse remodeling in heart failure and other cardiomyopathies. Endothelial-associated miRNAs, particularly miR-126, play essential roles in angiogenesis, vascular integrity, and endothelial repair [75]. Dysregulation of endothelial miRNAs has been demonstrated across cardiovascular disease states and may mechanistically underlie the impaired perfusion reserve observed in amyloid cardiomyopathy (Figure 3).

### 4.5. Distinct Pathobiological Mechanisms in AL and ATTR Cardiomyopathy: Implications for miRNA Regulation

Although AL and ATTR amyloidosis converge phenotypically toward restrictive cardiomyopathy, their underlying pathobiology differs fundamentally, and these differences are highly relevant when interpreting miRNA signaling patterns.

In AL amyloidosis, myocardial injury is driven not only by extracellular fibril deposition but also, and often predominantly, by direct proteotoxic effects of circulating amyloidogenic light chains [76]. Soluble light chain oligomers induce oxidative stress, mitochondrial dysfunction, calcium dysregulation, and activation of apoptotic signaling cascades within cardiomyocytes [4,45,46]. This toxic gain-of-function mechanism results in rapid cellular injury, early diastolic dysfunction, and frequently severe hemodynamic compromise even in the presence of modest amyloid burden. The biological environment in AL cardiomyopathy is therefore characterized by acute cellular stress, apoptosis, inflammatory activation, and metabolic disruption. In this context, miRNAs involved in regulating apoptosis, oxidative stress responses, mitochondrial homeostasis, and inflammatory signaling are likely to play a central role [71,72,73]. Dysregulation of apoptosis-associated miRNAs may reflect active cardiomyocyte injury and could potentially correlate with early disease severity and short-term prognosis.

In contrast, ATTR cardiomyopathy, particularly the wild-type form, typically follows a more indolent course characterized by progressive extracellular amyloid deposition and gradual myocardial stiffening. Imaging studies demonstrate diffuse myocardial infiltration and remodeling patterns distinct from AL cardiomyopathy [17]. Disease-modifying therapy trials further confirm the chronic progressive nature of ATTR cardiomyopathy, as shown in the ATTR-ACT study evaluating transthyretin stabilization [77]. Although cellular stress and mitochondrial dysfunction are also present, the dominant pathological process in ATTR appears to be chronic extracellular matrix remodeling and interstitial fibrosis. Activation of profibrotic signaling pathways, including TGF-β/Smad, MAPK, and Wnt/β-catenin cascades, promotes collagen types I and III deposition and progressive ventricular wall thickening [47,48,49,50]. Microvascular rarefaction and low-grade inflammation may further amplify fibrotic remodeling [43,44]. Within this framework, miRNAs that regulate fibroblast activation, collagen synthesis, and matrix degradation are likely to be particularly relevant [67,68,69,70]. Rather than reflecting acute cytotoxic injury, miRNA alterations in ATTR may correspond more closely to chronic remodeling and myocardial stiffening.

These mechanistic distinctions suggest that AL and ATTR cardiomyopathy may exhibit partially divergent miRNA signatures. In AL disease, miRNA profiles may be enriched for regulators of apoptosis, proteotoxic stress, and inflammatory pathways, whereas in ATTR cardiomyopathy, miRNAs governing extracellular matrix turnover and fibrotic signaling may predominate. However, direct comparative studies remain limited, and definitive disease-specific signatures have not yet been established.

Temporal dynamics further complicate interpretation. In early AL amyloidosis, miRNA changes may primarily reflect acute cardiomyocyte stress and reversible injury, whereas in advanced stages they may shift toward markers of fibrosis and structural remodeling. Conversely, in ATTR cardiomyopathy, early phases—particularly in asymptomatic mutation carriers—may exhibit subtle stress-related miRNA changes before overt hypertrophy or fibrosis is detectable by imaging. In advanced ATTR disease, miRNA profiles may predominantly mirror established extracellular matrix expansion and impaired ventricular compliance. Longitudinal studies are needed to determine whether distinct temporal miRNA trajectories exist and whether they can identify transition points between reversible dysfunction and irreversible structural remodeling.

Recognizing these biological differences is essential for interpreting circulating miRNA data and for designing future studies. Without careful phenotypic stratification and direct AL-versus-ATTR comparisons, observed miRNA alterations may reflect heterogeneous underlying mechanisms rather than disease-specific signatures. A more structured, mechanism-driven approach to miRNA investigation will therefore be critical for translating molecular findings into clinically meaningful biomarkers.

## 5. Circulating miRNAs as Noninvasive Biomarkers

Circulating miRNAs exhibit remarkable stability in peripheral blood, owing to their encapsulation within extracellular vesicles, association with lipoproteins, or binding to RNA-binding proteins [78]. Their tissue specificity and disease-related expression patterns make them attractive candidates for biomarker development in cardiovascular disorders, including cardiomyopathies and heart failure [64]. In this context, we have shown that specific circulating miRNAs, including miR-21 and miR-1, are differentially expressed in cardiovascular disease states and correlate with indices of myocardial injury and functional impairment, supporting their potential role as clinically informative blood-based biomarkers [79,80].

In CA, dysregulated miRNA expression reflects fundamental pathogenic mechanisms such as amyloid deposition, cardiomyocyte stress, fibrosis, and inflammation. Fibrosis-associated miRNAs, including miR-21 and members of the miR-29 family, have been implicated in extracellular matrix remodeling and myocardial stiffening—key pathological features of amyloid cardiomyopathy [67,70]. These circulating miRNAs may provide complementary information to established cardiac biomarkers such as N-terminal pro-B-type natriuretic peptide (NT-proBNP) and cardiac troponins, which reflect hemodynamic stress and myocardial injury but lack disease specificity.

### 5.1. miRNAs for Differentiating Amyloidosis Subtypes

Distinguishing between light-chain (AL) amyloidosis and ATTR amyloidosis is of paramount clinical importance, as prognosis, therapeutic strategies, and urgency of treatment differ substantially between the two entities [9]. Emerging evidence suggests that miRNA expression profiles may differ between amyloidosis subtypes, reflecting distinct underlying biological mechanisms. AL amyloidosis, driven by plasma cell dyscrasia and systemic proteotoxicity, has been associated with dysregulation of miRNAs involved in immune responses, apoptosis, and oxidative stress, including miR-150 and miR-155 [69]. In contrast, ATTR amyloidosis—characterized by chronic myocardial infiltration and progressive fibrosis—appears to be associated with miRNAs linked to extracellular matrix remodeling and cardiomyocyte stress signaling [8,70]. A comprehensive profiling study of circulating microRNAs in patients with transthyretin variant amyloidosis (ATTRv) identified substantial dysregulation compared with multiple control groups. Specifically, ATTRv patients exhibited 33 up-regulated and 48 down-regulated miRNAs relative to healthy controls, 9 up-regulated and 30 down-regulated miRNAs compared with patients with Charcot–Marie–Tooth disease (CMT), and 19 up-regulated and 38 down-regulated miRNAs compared with asymptomatic carriers of transthyretin variants (TTRv) [62]. Subsequent validation analyses highlighted miR-150-5p as a robust biomarker capable of discriminating symptomatic ATTRv patients from asymptomatic TTRv carriers (47). Functional studies using Schwann cell culture models demonstrated that miR-150-5p acts as a potent negative regulator of CREB, BDNF, and NGF expression—genes that are also implicated in cardiac dysfunction [62]. In a study investigating microRNA dysregulation in AL amyloidosis, bone marrow plasma cells (BMPCs) were purified from patients with AL amyloidosis and from control subjects. Comparative microarray analysis revealed that ten miRNAs were up-regulated by more than 1.5-fold in AL amyloidosis. These findings were subsequently validated by stem-loop RT-qPCR for the most markedly up-regulated miRNAs, including miR-148a, miR-26a, and miR-16. Notably, miR-16—previously implicated in other hematopoietic malignancies—was significantly elevated in AL patients at diagnosis and in treated individuals with persistent monoclonal plasma cells in the bone marrow, but not in patients who achieved hematologic remission following therapy. Collectively, these results indicate that miRNA expression is dysregulated in clonal plasma cells in AL amyloidosis and suggest that specific miRNAs may serve as potential biomarkers of disease presence and treatment response [81]. Although current data are limited and largely exploratory, these findings support the concept that miRNA signatures may assist in subtype discrimination when conventional diagnostic algorithms are inconclusive. A summary of key miRNAs implicated in cardiac amyloidosis, their associated molecular pathways, and the current level of supporting evidence is provided in Table 1.

This table summarizes the principal microRNAs implicated in the pathobiology of cardiac amyloidosis, integrating both circulating biomarker data and mechanistic pathway evidence. The listed miRNAs cluster around four dominant biological processes central to amyloid-induced myocardial injury: (i) fibrotic remodeling and extracellular matrix regulation (e.g., miR-21, miR-29 family), (ii) apoptosis and proteotoxic stress signaling (e.g., miR-34a, miR-320a), (iii) inflammatory and immune activation pathways (e.g., miR-155, miR-146a, miR-181b), and (iv) endothelial dysfunction and vascular homeostasis (e.g., miR-126, miR-223).

In AL amyloidosis, miRNAs related to plasma-cell biology and proteotoxic stress appear particularly relevant, whereas in ATTR cardiomyopathy, miRNAs associated with chronic fibrotic remodeling and TGF-β/Smad-mediated signaling predominate. While several circulating miRNAs have demonstrated potential diagnostic discrimination in small human cohorts, much of the mechanistic framework derives from broader cardiovascular research rather than amyloid-specific experimental validation. Accordingly, the level of evidence varies across entries, underscoring the need for larger, well-characterized studies to clarify disease specificity and clinical applicability.

### 5.2. Diagnostic Value in Early and Subclinical Disease

One of the most promising potential applications of miRNAs in cardiac amyloidosis lies in the detection of early or subclinical cardiac involvement. Structural and functional abnormalities often develop gradually and may remain undetected by conventional imaging modalities until advanced disease stages [7,32]. In contrast, miRNA dysregulation may occur earlier in the disease course, reflecting molecular alterations that precede overt myocardial dysfunction.

Consistent with this concept, we have shown that alterations in circulating miRNAs associated with myocardial remodeling and vascular dysfunction can be detected in cardiovascular conditions prior to the development of overt structural heart disease, supporting their potential utility as early diagnostic or risk-stratification marker [79,80,82]. These observations align with broader evidence suggesting that stress- and fibrosis-related miRNAs may be altered before significant echocardiographic or magnetic resonance abnormalities become apparent [68]. Applied to cardiac amyloidosis, these findings indicate that miRNA-based assays could support earlier recognition of cardiac involvement, particularly among individuals at increased risk or in preclinical stages of disease. In clinical practice, circulating miRNAs are unlikely to replace established diagnostic tools but may add incremental value when incorporated into multimodal diagnostic pathways. Integration of miRNA profiling with echocardiography, cardiac magnetic resonance imaging, nuclear scintigraphy, and conventional serum biomarkers could facilitate earlier referral, improve diagnostic confidence, and enable more accurate disease classification and monitoring [10,11]. Such integrative approaches could reduce reliance on invasive endomyocardial biopsy in selected cases and improve diagnostic confidence, particularly in early or atypical presentations.

However, methodological challenges—including variability in miRNA isolation techniques, normalization strategies, and analytical platforms—currently limit direct clinical translation [78]. Despite growing interest, miRNA-based diagnostics in CA remain at an early stage of development. Most available studies are observational, involve small patient cohorts, and use heterogeneous methodologies. Furthermore, overlap in miRNA dysregulation across different cardiovascular diseases may limit disease specificity when individual miRNAs are considered in isolation [64]. Future research should prioritize the development and validation of multi-miRNA diagnostic panels, longitudinal studies assessing temporal changes in miRNA expression, and standardized protocols for miRNA quantification. Advances in high-throughput sequencing, bioinformatics, and machine learning are expected to facilitate the identification of robust diagnostic signatures and accelerate the clinical translation of miRNA-based diagnostics in cardiac amyloidosis.

### 5.3. Clinical Integration of Circulating miRNAs in Cardiac Amyloidosis

Although numerous studies have described altered circulating miRNA profiles in cardiac amyloidosis, their potential role within established diagnostic and therapeutic frameworks requires clearer definition. At present, miRNAs should not be viewed as standalone diagnostic tools but rather as potential complementary biomarkers within a multimodal approach.

The current diagnostic pathway for suspected cardiac amyloidosis relies on a combination of clinical suspicion, cardiac imaging, laboratory biomarkers, and, when necessary, tissue confirmation. Bone scintigraphy has become central for noninvasive diagnosis of ATTR cardiomyopathy in the absence of monoclonal gammopathy [10], whereas cardiac magnetic resonance imaging provides structural and tissue characterization data. Circulating biomarkers such as NT-proBNP and cardiac troponins are used for staging and prognostic stratification in both AL and ATTR amyloidosis [83]. Within this framework, circulating miRNAs could theoretically serve three potential functions: early detection, disease phenotyping, and treatment-response monitoring.

First, miRNAs may have value in identifying subclinical myocardial involvement, particularly in at-risk populations such as carriers of pathogenic transthyretin variants or patients with monoclonal gammopathy of undetermined significance. Because miRNAs reflect active molecular signaling, they could potentially detect early cellular stress before overt structural abnormalities become apparent on imaging. However, this hypothesis remains unproven, as no prospective screening studies have validated miRNA panels for early detection.

Second, miRNAs may assist in disease phenotyping. Given the mechanistic differences between AL and ATTR cardiomyopathy, disease-specific miRNA signatures could theoretically complement existing diagnostic algorithms, particularly in equivocal cases. For example, miRNAs associated with apoptosis and proteotoxic stress might support suspicion of AL-related cardiotoxicity, whereas fibrosis-associated miRNAs might be more characteristic of ATTR-related remodeling. At present, however, no miRNA signature has demonstrated sufficient sensitivity, specificity, or incremental diagnostic value beyond established modalities to justify routine clinical use.

Third, circulating miRNAs may have potential as dynamic biomarkers of therapeutic response. In AL amyloidosis, rapid hematologic response to chemotherapy is closely linked to cardiac recovery, yet early cardiac response markers remain limited [84]. If specific miRNAs reflect ongoing cardiomyocyte injury, their normalization could theoretically precede structural improvement. Similarly, in ATTR cardiomyopathy, disease-modifying therapies such as transthyretin stabilizers or gene-silencing agents may alter molecular remodeling pathways before measurable changes occur in wall thickness or extracellular volume. Preliminary studies suggest that circulating miRNA levels may change following treatment, but these findings require validation in larger longitudinal cohorts with standardized sampling protocols.

Importantly, integration of miRNAs into clinical practice would require demonstration of clear incremental value over existing biomarkers. Any proposed miRNA panel must show reproducibility, robust discrimination between disease states, and additive prognostic or diagnostic information beyond NT-proBNP, troponins, and advanced imaging parameters. Cost-effectiveness, assay standardization, and turnaround time would also need consideration before implementation.

Therefore, while circulating miRNAs represent promising molecular indicators of myocardial stress and remodeling in cardiac amyloidosis, their current role remains investigational. Carefully designed prospective studies incorporating imaging, conventional biomarkers, and clinical outcomes are essential to determine whether miRNA profiling can meaningfully enhance diagnostic precision, risk stratification, or therapeutic monitoring.

## 6. Therapeutic Applications of MicroRNAs in Cardiac Amyloidosis

Beyond their diagnostic and prognostic potential, microRNAs have attracted increasing interest as potential therapeutic targets in cardiac amyloidosis. By regulating multiple genes within interconnected signaling pathways, miRNAs occupy a central position in the molecular networks governing amyloid-induced myocardial stress, fibrosis, inflammation, and cardiomyocyte dysfunction. Modulating miRNA activity therefore represents a theoretically attractive strategy to influence disease progression at a post-transcriptional level.

The pathophysiology of CA is multifactorial, involving extracellular amyloid deposition, mechanical myocardial stiffening, proteotoxic stress, oxidative injury, and progressive fibrotic remodeling. Conventional therapies largely target upstream processes—such as suppression of light-chain production in AL amyloidosis or stabilization and silencing of transthyretin in ATTR amyloidosis—while downstream myocardial remodeling often continues despite effective disease-modifying treatment. MiRNAs offer a complementary therapeutic avenue by modulating intracellular responses to amyloid toxicity. Several miRNAs implicated in fibrosis (e.g., miR-21, miR-29), cardiomyocyte survival (e.g., miR-133, miR-1), and inflammatory signaling have been shown to influence pathways relevant to myocardial remodeling and diastolic dysfunction [64,67,68,70]. Given their pleiotropic effects, therapeutic modulation of miRNAs could theoretically attenuate maladaptive remodeling processes common to both AL and ATT R cardiomyopathy.

Myocardial fibrosis is a central determinant of diastolic dysfunction, arrhythmogenesis, and adverse prognosis in CA. Among the best-studied miRNAs in cardiac fibrosis, miR-21 has been shown to promote fibroblast activation and extracellular matrix deposition through MAP kinase and TGF-β-dependent pathways [67]. Experimental inhibition of miR-21 has been associated with attenuation of cardiac fibrosis and improved myocardial compliance in preclinical models [85]. Conversely, members of the miR-29 family function as negative regulators of collagen and extracellular matrix gene expression, and their downregulation has been linked to fibrotic remodeling in multiple cardiac disease states [70]. Restoring miR-29 expression has demonstrated antifibrotic effects in experimental settings, suggesting potential therapeutic relevance for infiltrative cardiomyopathies characterized by progressive myocardial stiffening.

Although these findings are not specific to amyloidosis, the shared fibrotic phenotype supports cautious extrapolation to CA, particularly in ATTR cardiomyopathy, where fibrosis evolves over a prolonged disease course.

Several platforms for miRNA modulation have been developed, including antisense oligonucleotides (antagomiRs), miRNA mimics, and locked nucleic acid (LNA)-based inhibitors. These approaches have entered clinical testing in noncardiac diseases, demonstrating the feasibility of targeting miRNAs in humans [86]. However, translation to cardiovascular disease—and particularly to CA—faces substantial challenges. Key obstacles include tissue-specific delivery to the myocardium, off-target effects due to the pleiotropic nature of miRNAs, immune activation, and long-term safety concerns. Furthermore, the advanced age and comorbidity burden typical of patients with ATTR amyloidosis necessitate particularly stringent safety profiles for any novel therapeutic intervention.

Rather than replacing established disease-modifying treatments, miRNA-based therapies—if successfully developed—are more likely to function as adjunctive strategies. In AL amyloidosis, miRNA modulation could potentially complement plasma cell-directed therapies by mitigating downstream myocardial remodeling. In ATTR amyloidosis, miRNA-targeted approaches might synergize with transthyretin stabilizers or gene-silencing therapies by attenuating fibrosis and cardiomyocyte stress [77,87].

Such combination strategies align with a precision-medicine framework, in which upstream amyloidogenic processes and downstream myocardial responses are addressed simultaneously.

## 7. Critical Gaps, Unresolved Controversies, and Future Research Priorities

Despite increasing scientific interest in microRNAs in cardiac amyloidosis, the field remains at an early translational stage, and several conceptual and methodological limitations continue to restrict clinical applicability. While numerous studies suggest mechanistic and biomarker potential, the overall level of evidence remains heterogeneous and, in many cases, preliminary.

Several aspects of miRNA involvement in amyloid cardiomyopathy can be considered relatively well established. It is widely accepted that miRNAs regulate key signaling pathways implicated in apoptosis, oxidative stress, inflammation, and extracellular matrix remodeling, all of which are central to the pathophysiology of amyloid-induced myocardial injury [88,89]. Distinct circulating miRNA profiles have been reported in patients with cardiac amyloidosis compared with non-amyloid heart failure and healthy controls. In AL amyloidosis, proteotoxic light chains activate stress-related signaling cascades that are known to be under miRNA control, whereas in ATTR cardiomyopathy, progressive fibrotic remodeling involves canonical TGF-β/Smad and MAPK pathways, also subject to post-transcriptional regulation by miRNAs [90]. However, much of this mechanistic framework derives from broader cardiovascular research, and direct validation in well-characterized amyloidosis cohorts remains limited.

Emerging evidence suggests that circulating miRNA signatures may differ between AL and ATTR cardiomyopathy, potentially reflecting their distinct pathogenic mechanisms [61,62]. Preliminary data suggest that specific circulating miRNAs may correlate with established markers of disease severity such as NT-proBNP and measures of cardiac structure and function in heart failure contexts, [91,92]. Small clinical studies in AL amyloidosis have also demonstrated longitudinal changes in circulating miRNAs following effective therapy [81]. Although these findings are promising, they are derived predominantly from small, single-center cohorts and lack independent external validation, underscoring the need for larger, multicenter validation studies.

At present, several important questions remain unresolved. No circulating miRNA panel has been prospectively validated for diagnostic use in cardiac amyloidosis, nor has any demonstrated clear incremental value over established modalities such as bone scintigraphy, cardiac magnetic resonance, or established biomarkers including NT-proBNP and troponins. Similarly, robust prognostic stratification independent of established staging systems has not been consistently demonstrated. The temporal dynamics of miRNA expression across disease stages, particularly during the early preclinical phase of ATTR amyloidosis compared with advanced restrictive cardiomyopathy, are insufficiently characterized. Whether specific miRNA signatures reflect reversible myocardial injury or irreversible fibrotic remodeling also remains uncertain.

Methodological heterogeneity further complicates interpretation. Variability in plasma or serum processing, RNA extraction protocols, normalization strategies, and analytical platforms significantly limits reproducibility across studies [93]. Differences between quantitative PCR, microarray-based profiling, and next-generation sequencing approaches introduce additional variability. Sample sizes are frequently small, and direct comparative analyses between AL and ATTR cardiomyopathy are scarce, despite their fundamentally distinct biological mechanisms.

Future research should therefore prioritize adequately powered multicenter validation studies using standardized pre-analytical and analytical protocols. Direct head-to-head comparisons between AL and ATTR cardiomyopathy are necessary to clarify disease-specific miRNA signatures. Longitudinal studies evaluating miRNA dynamics during disease progression and in response to therapy will be essential to determine clinical utility. Integration of miRNA profiling with proteomic, imaging, and conventional biomarker data may allow development of multimodal diagnostic and prognostic models. Ultimately, only rigorous validation and harmonization of methodology will allow miRNAs to transition from mechanistic correlates to clinically actionable biomarkers in cardiac amyloidosis.

## 8. Future Directions and Challenges

Despite growing enthusiasm, several challenges must be addressed before miRNA-based approaches can be integrated into routine clinical practice. Methodological heterogeneity in miRNA detection platforms, normalization strategies, and study design limits comparability across cohorts. Standardization of pre-analytical and analytical workflows is essential to enable validation and regulatory approval [64,68]. Additionally, the causal versus associative roles of specific miRNAs in amyloid-related cardiac dysfunction remain incompletely defined. Most available data are observational, underscoring the need for mechanistic studies and prospective clinical trials to clarify therapeutic relevance. Given the systemic nature of amyloidosis, careful consideration must also be given to off-target effects and interactions between cardiac and extracardiac miRNA signaling.

Future research should focus on integrating miRNA profiling with established imaging and biochemical markers to develop multimodal risk stratification strategies. Large, well-characterized cohorts with longitudinal follow-up will be critical to defining clinically meaningful miRNA signatures and thresholds. Advances in delivery technologies and improved understanding of miRNA biology may ultimately enable precision targeting of pathogenic pathways in amyloid-related heart failure.

Ultimately, the clinical integration of miRNA-based approaches will depend on demonstrating clear incremental value over established diagnostic and therapeutic strategies. For diagnostics, this includes improved early detection, subtype differentiation, or risk stratification. For therapeutics, it requires evidence of meaningful clinical benefit, acceptable safety profiles, and feasibility within real-world healthcare settings [11,68]. Multidisciplinary collaboration between clinicians, molecular scientists, and data scientists will be essential to translate miRNA research into clinically actionable tools for patients with cardiac amyloidosis.

## 9. Conclusions

CA represents a complex and heterogeneous cause of heart failure, driven by distinct yet overlapping mechanisms in transthyretin and light-chain disease. MicroRNAs have emerged as central regulators of key pathways underlying amyloid-related myocardial dysfunction, including fibrosis, mitochondrial failure, oxidative stress, inflammation, and microvascular impairment. Accumulating evidence supports the potential of circulating and tissue miRNA signatures to enhance diagnostic accuracy, enable etiologic differentiation, and refine prognostic assessment beyond conventional biomarkers.

As RNA-based therapeutics continue to advance, miRNAs offer a compelling bridge between mechanistic insight and clinical translation in cardiac amyloidosis. Integration of miRNA profiling with contemporary multimodality imaging and disease-specific therapies may facilitate earlier diagnosis and support precision management of amyloid-related heart failure. Continued translational and clinical investigation will be essential to realize the full potential of miRNA-based strategies in this rapidly evolving field.

## Figures and Tables

**Table 1:** Key microRNAs implicated in cardiac amyloidosis: molecular pathways and level of evidence.

microRNA	Primary Molecular Pathway(s)	Principal Biological Effect	Amyloidosis Subtype	Level of Evidence	Key Reference(s)
miR-339-3p	Disease-associated circulating signature	Diagnostic discrimination	AL, ATTR	Human blood profiling	[61]
miR-150-5p	CREB, BDNF, NGF regulation	Differentiates symptomatic ATTRv from asymptomatic carriers; modulates neuro-cardiac signaling	ATTRv	Human circulating miRNA profiling + functional validation	[62]
miR-21	TGF-β/Smad signaling, fibroblast activation	Promotes myocardial fibrosis and ECM expansion	ATTR (predominant)	Strong cardiovascular evidence; indirect amyloidosis relevance	[67]
miR-29 family	ECM regulation (collagen I/III, fibrillin)	Anti-fibrotic; downregulation promotes myocardial stiffness	AL, ATTR	Strong cardiovascular and experimental evidence	[70]
miR-199a	Fibroblast proliferation, hypertrophic signaling	Enhances fibrotic remodeling	ATTR	Experimental cardiovascular evidence	[68]
miR-155	Inflammatory and immune signaling	Pro-inflammatory and pro-fibrotic effects	ATTR (putative)	Indirect cardiovascular evidence	[69]
miR-34a	Apoptosis, aging-related pathways	Promotes cardiomyocyte apoptosis	AL (putative)	Experimental cardiovascular evidence	[71]
miR-320a	Oxidative stress, mitochondrial dysfunction	Induces cardiomyocyte injury and apoptosis	AL (putative)	Experimental cardiovascular evidence	[72]
miR-181b	Oxidative stress and inflammatory signaling	Regulates cardiomyocyte stress responses	AL (putative)	Experimental cardiovascular evidence	[73]
miR-146a	NF-κB-mediated innate immune signaling	Modulates inflammatory stress responses	ATTR (putative)	Experimental cardiovascular evidence	[74]
miR-126	Endothelial integrity and angiogenesis	Maintains vascular homeostasis	AL, ATTR	Strong endothelial cardiovascular evidence	[75]
miR-223	Inflammation, platelet–endothelial crosstalk	Modulates vascular inflammation	AL, ATTR	Indirect cardiovascular evidence	[69]
miR-148a	Plasma-cell differentiation, immunoglobulin synthesis	Upregulated in clonal plasma cells	AL	Human bone marrow plasma-cell profiling	[81]
miR-26a	Cell-cycle regulation, plasma-cell survival	Associated with clonal plasma-cell persistence	AL	Human bone marrow plasma-cell profiling	[81]
miR-16	Apoptosis and hematopoietic regulation	Elevated in active AL and persistent monoclonal plasma cells; normalizes in remission	AL	Human bone marrow plasma-cell profiling	[81]

Abbreviations: AL, immunoglobulin light-chain amyloidosis; ATTR, transthyretin amyloidosis; ATTRv, variant transthyretin amyloidosis; ECM, extracellular matrix; CREB, cAMP response element-binding protein; BDNF, Brain-derived neurotrophic factor; NGF, Nerve growth factor; TGF-β, Transforming growth factor beta; Smad, Small mothers against decapentaplegic (SMAD family proteins).

## Data Availability

No new data were generated or analyzed in this study. Data sharing is not applicable to this article. This manuscript is a narrative review and does not contain original experimental data, patient data, or biological images. The schematic figures and graphical abstract were conceptually designed by the authors to summarize mechanisms described in the literature. An artificial intelligence (AI) language model (ChatGPT, OpenAI; GPT-5.3) was used to assist with language refinement and organizational structuring of figure concepts. No AI tools were used to generate scientific data, biological images, experimental results, or novel interpretations. All figures were assembled and finalized by the authors using licensed scientific illustration software. The authors take full responsibility for the accuracy, originality, and scientific integrity of all graphical content.

## References

[1] Gill S.S., Fellin E., Stampke L., Zhao Y., Masri A. (2022). Clinical Clues and Diagnostic Workup of Cardiac Amyloidosis. Methodis. Debakey Cardiovasc. J..

[2] Wechalekar A.D., Gillmore J.D., Hawkins P.N. (2016). Systemic amyloidosis. Lancet.

[3] Buxbaum J.N., Eisenberg D.S., Fändrich M., McPhail E.D., Merlini G., Saraiva M.J.M., Sekijima Y., Westermark P. (2024). Amyloid nomenclature 2024: Update, novel proteins, and recommendations by the International Society of Amyloidosis (ISA) Nomenclature Committee. Amyloid.

[4] Brenner D.A., Jain M., Pimentel D.R., Wang B., Connors L.H., Skinner M., Apstein C.S., Liao R. (2004). Human amyloidogenic light chains directly impair cardiomyocyte function through an increase in cellular oxidant stress. Circ. Res..

[5] Falk R.H., Alexander K.M., Liao R., Dorbala S. (2016). AL (Light-Chain) Cardiac Amyloidosis: A Review of Diagnosis and Therapy. J. Am. Coll. Cardiol..

[6] Kittleson M.M., Maurer M.S., Ambardekar A.V., Bullock-Palmer R.P., Chang P.P., Eisen H.J., Nair A.P., Nativi-Nicolau J., Ruberg F.L., American Heart Association Heart Failure and Transplantation Committee of the Council on Clinical Cardiology (2020). Cardiac Amyloidosis: Evolving Diagnosis and Management: A Scientific Statement from the American Heart Association. Circulation.

[7] Martinez-Naharro A., Hawkins P.N., Fontana M. (2018). Cardiac amyloidosis. Clin. Med..

[8] Rapezzi C., Quarta C.C., Riva L., Longhi S., Gallelli I., Lorenzini M., Ciliberti P., Biagini E., Salvi F., Branzi A. (2010). Transthyretin-related amyloidoses and the heart: A clinical overview. Nat. Rev. Cardiol..

[9] Maurer M.S., Elliott P., Merlini G., Shah S.J., Cruz M.W., Flynn A., Gundapaneni B., Hahn C., Riley S., Schwartz J. (2017). Design and Rationale of the Phase 3 ATTR-ACT Clinical Trial (Tafamidis in Transthyretin Cardiomyopathy Clinical Trial). Circ. Heart Fail..

[10] Gillmore J.D., Maurer M.S., Falk R.H., Merlini G., Damy T., Dispenzieri A., Wechalekar A.D., Berk J.L., Quarta C.C., Grogan M. (2016). Nonbiopsy Diagnosis of Cardiac Transthyretin Amyloidosis. Circulation.

[11] Dorbala S., Cuddy S., Falk R.H. (2020). How to Image Cardiac Amyloidosis: A Practical Approach. JACC Cardiovasc. Imaging.

[12] Thum T. (2014). Noncoding RNAs and myocardial fibrosis. Nat. Rev. Cardiol..

[13] Bauersachs J., Thum T. (2011). Biogenesis and regulation of cardiovascular microRNAs. Circ. Res..

[14] Siasos G., Bletsa E., Stampouloglou P.K., Oikonomou E., Tsigkou V., Paschou S.A., Vlasis K., Marinos G., Vavuranakis M., Stefanadis C. (2020). MicroRNAs in cardiovascular disease. Hell. J. Cardiol..

[15] Johnson S.M., Connelly S., Fearns C., Powers E.T., Kelly J.W. (2012). The transthyretin amyloidoses: From delineating the molecular mechanism of aggregation linked to pathology to a regulatory-agency-approved drug. J. Mol. Biol..

[16] Quintas A., Saraiva M.J.M., Brito R.M.M. (1997). The amyloidogenic potential of transthyretin variants correlates with their tendency to aggregate in solution. FEBS Lett..

[17] Ruberg F.L., Berk J.L. (2012). Transthyretin (TTR) Cardiac Amyloidosis. Circulation.

[18] Kazman P., Absmeier R.M., Engelhardt H., Buchner J. (2021). Dissection of the amyloid formation pathway in AL amyloidosis. Nat. Commun..

[19] Poshusta T.L., Sikkink L.A., Leung N., Clark R.J., Dispenzieri A., Ramirez-Alvarado M. (2009). Mutations in Specific Structural Regions of Immunoglobulin Light Chains Are Associated with Free Light Chain Levels in Patients with AL Amyloidosis. PLoS ONE.

[20] Zvida-Bloch T., Muchtar E., Dispenzieri A., Shpilberg O., Hershkovitz-Rokah O. (2025). The molecular landscape of AL amyloidosis. Br. J. Haematol..

[21] Imperlini E., Gnecchi M., Rognoni P., Sabidò E., Ciuffreda M.C., Palladini G., Espadas G., Mancuso F.M., Bozzola M., Malpasso G. (2017). Proteotoxicity in cardiac amyloidosis: Amyloidogenic light chains affect the levels of intracellular proteins in human heart cells. Sci. Rep..

[22] Bézard M., Vartanian-Grimaldi J.-S., Henri J., Calin D., Zaroui A., Kharoubi M., Damy T., Agbulut O., Kordeli E. (2025). Amyloidogenic immunoglobulin light chains disturb contractile function and calcium transients in a human cardiac spheroid model of light chain (AL) amyloidosis. Sci. Rep..

[23] Merlini G., Bellotti V. (2003). Molecular mechanisms of amyloidosis. N. Engl. J. Med..

[24] Liao R., Jain M., Teller P., Connors L.H., Ngoy S., Skinner M., Falk R.H., Apstein C.S. (2001). Infusion of Light Chains from Patients with Cardiac Amyloidosis Causes Diastolic Dysfunction in Isolated Mouse Hearts. Circulation.

[25] Dittloff K.T., Spanghero E., Solís C., Banach K., Russell B. (2022). Transthyretin deposition alters cardiomyocyte sarcomeric architecture, calcium transients, and contractile force. Physiol. Rep..

[26] Papathanasiou M., Schlender L.S., Johnson V.L., Wakili R. (2024). Arrhythmias and amyloidosis. Herzschrittmacherther Elektrophysiol..

[27] Vergaro G., Aimo A., Rapezzi C., Castiglione V., Fabiani I., Pucci A., Buda G., Passino C., Lupón J., Bayes-Genis A. (2022). Atrial Amyloidosis: Mechanisms and Clinical Manifestations. Eur. J. Heart Fail..

[28] Holcman K., Ząbek A., Boczar K., Podolec P., Kostkiewicz M. (2024). Management of Arrhythmias and Conduction Disorders in Amyloid Cardiomyopathy. J. Clin. Med..

[29] Liao J., Pan X. (2025). Systemic light chain cardiac amyloidosis with atrioventricular block. Front. Cardiovasc. Med..

[30] Migrino R.Q., Truran S., Gutterman D.D., Franco D.A., Bright M., Schlundt B., Timmons M., Motta A., Phillips S.A., Hari P. (2011). Human microvascular dysfunction and apoptotic injury induced by AL amyloidosis light chain proteins. Am. J. Physiol. Heart Circ. Physiol..

[31] Dorbala S., Vangala D., Bruyere J., Quarta C., Kruger J., Padera R., Foster C., Hanley M., Di Carli M.F., Falk R. (2014). Coronary microvascular dysfunction is related to abnormalities in myocardial structure and function in cardiac amyloidosis. JACC Heart Fail..

[32] Fontana M., Pica S., Reant P., Abdel-Gadir A., Treibel T.A., Banypersad S.M., Maestrini V., Barcella W., Rosmini S., Bulluck H. (2015). Prognostic Value of Late Gadolinium Enhancement Cardiovascular Magnetic Resonance in Cardiac Amyloidosis. Circulation.

[33] Nakagawa M., Sekijima Y., Yazaki M., Tojo K., Yoshinaga T., Doden T., Koyama J., Yanagisawa S., Ikeda S. (2016). Carpal tunnel syndrome: A common initial symptom of systemic wild-type ATTR (ATTRwt) amyloidosis. Amyloid.

[34] Takashio S., Nishi M., Tsuruta Y., Tsujita K. (2020). Wild-type transthyretin amyloid cardiomyopathy complicated by spinal canal stenosis, carpal tunnel syndrome, and rotator cuff tears: A case report. Eur. Heart J.-Case Rep..

[35] Geller H.I., Singh A., Alexander K.M., Mirto T.M., Falk R.H. (2017). Association Between Ruptured Distal Biceps Tendon and Wild-Type Transthyretin Cardiac Amyloidosis. JAMA.

[36] Perfetto F., Zampieri M., Bandini G., Fedi R., Tarquini R., Santi R., Novelli L., Allinovi M., Argirò A., Cappelli F. (2022). Transthyretin Cardiac Amyloidosis: A Cardio-Orthopedic Disease. Biomedicines.

[37] Tschöpe C., Elsanhoury A., Kristen A.V. (2025). Transthyretin Amyloid Cardiomyopathy-2025 Update: Current Diagnostic Approaches and Emerging Therapeutic Options. J. Clin. Med..

[38] Karam C., Moffit C., Summers C., Merkel M.P., Kochman F.M., Weijers L., Puls M., Schurer M., Jones E., Mason N. (2024). The journey to diagnosis of wild-type transthyretin-mediated (ATTRwt) amyloidosis: A path with multisystem involvement. Orphanet J. Rare Dis..

[39] Adams D., Sekijima Y., Conceição I., Waddington-Cruz M., Polydefkis M., Echaniz-Laguna A., Reilly M.M. (2023). Hereditary transthyretin amyloid neuropathies: Advances in pathophysiology, biomarkers, and treatment. Lancet Neurol..

[40] Ladefoged B., Pedersen A.D., Seefeldt J., Nielsen B.R.R., Eiskjær H., Lichscheidt E., Clemmensen T., Gillmore J.D., Poulsen S.H. (2024). Exercise Hemodynamics and Mitochondrial Oxidative Capacity in Disease Stages of Wild-Type Transthyretin Amyloid Cardiomyopathy. J. Am. Heart Assoc..

[41] Fan Y.Z., Qiu Z.P., Wang Z.Y., Yang W.B., Huang F.Y., Chen Y.J., Yang K., Jin W. (2023). Vitronectin promotes TTR deposition and mitochondrial damage in ATTR-CA mice. Eur. Heart J..

[42] Netzel B.C., Charlesworth M.C., Johnson K.L., French A.J., Dispenzieri A., Maleszewski J.J., McPhail E.D., Grogan M., Redfield M.M., Weivoda M. (2025). Whole tissue proteomic analyses of cardiac ATTR and AL unveil mechanisms of tissue damage. Amyloid.

[43] Phua T.J. (2023). Understanding human aging and the fundamental cell signaling link in age-related diseases: The middle-aging hypovascularity hypoxia hypothesis. Front. Aging.

[44] Russo M.A., Tomino C., Vernucci E., Limana F., Sansone L., Frustaci A., Tafani M. (2019). Hypoxia and Inflammation as a Consequence of β-Fibril Accumulation: A Perspective View for New Potential Therapeutic Targets. Oxidative Med. Cell Longev..

[45] Diomede L., Romeo M., Rognoni P., Beeg M., Foray C., Ghibaudi E., Palladini G., Cherny R.A., Verga L., Capello G.L. (2017). Cardiac Light Chain Amyloidosis: The Role of Metal Ions in Oxidative Stress and Mitochondrial Damage. Antioxid. Redox Signal..

[46] Shi J., Guan J., Jiang B., Brenner D.A., Del Monte F., Ward J.E., Connors L.H., Sawyer D.B., Semigran M.J., Macgillivray T.E. (2010). Amyloidogenic light chains induce cardiomyocyte contractile dysfunction and apoptosis via a non-canonical p38alpha MAPK pathway. Proc. Natl. Acad. Sci. USA.

[47] Dobaczewski M., Bujak M., Li N., Gonzalez-Quesada C., Mendoza L.H., Wang X.F., Frangogiannis N.G. (2010). Smad3 signaling critically regulates fibroblast phenotype and function in healing myocardial infarction. Circ. Res..

[48] Baba Y., Maezawa Y., Kondo N., Minamizuka T., Udagawa H., Ide S., Ide K., Teramoto N., Yamaguchi A., Kaneko H. (2025). Tcf21 modulates fibroblast activation and promotes cardiac fibrosis after injury via Pdgfrb signaling. Sci. Rep..

[49] Frangogiannis N.G. (2021). Cardiac fibrosis. Cardiovasc. Res..

[50] Yousefi F., Shabaninejad Z., Vakili S., Derakhshan M., Movahedpour A., Dabiri H., Ghasemi Y., Mahjoubin-Tehran M., Nikoozadeh A., Savardashtaki A. (2020). TGF-β and WNT signaling pathways in cardiac fibrosis: Non-coding RNAs come into focus. Cell Commun. Signal..

[51] Zhang Y., Huang X.R., Wei L.H., Chung A.C., Yu C.M., Lan H.Y. (2014). miR-29b as a therapeutic agent for angiotensin II-induced cardiac fibrosis by targeting TGF-β/Smad3 signaling. Mol. Ther..

[52] Zhao X., Wang K., Liao Y., Zeng Q., Li Y., Hu F., Liu Y., Meng K., Qian C., Zhang Q. (2015). MicroRNA-101a inhibits cardiac fibrosis induced by hypoxia via targeting TGFβRI on cardiac fibroblasts. Cell Physiol. Biochem..

[53] Yousefi F., Soltani B.M., Rabbani S. (2021). MicroRNA-331 inhibits isoproterenol-induced expression of profibrotic genes in cardiac myofibroblasts via the TGFβ/smad3 signaling pathway. Sci. Rep..

[54] Morfino P., Aimo A., Franzini M., Vergaro G., Castiglione V., Panichella G., Limongelli G., Emdin M. (2024). Pathophysiology of Cardiac Amyloidosis. Heart Fail. Clin..

[55] Ong S.B., Kalkhoran S.B., Hernández-Reséndiz S., Samangouei P., Ong S.G., Hausenloy D.J. (2017). Mitochondrial-Shaping Proteins in Cardiac Health and Disease—The Long and the Short of It!. Cardiovasc. Drugs Ther..

[56] Gustafsson Å.B., Dorn G.W. (2019). Evolving and Expanding the Roles of Mitophagy as a Homeostatic and Pathogenic Process. Physiol. Rev..

[57] Kubli D.A., Gustafsson Å.B. (2012). Mitochondria and mitophagy: The yin and yang of cell death control. Circ. Res..

[58] Arany Z., He H., Lin J., Hoyer K., Handschin C., Toka O., Ahmad F., Matsui T., Chin S., Wu P.H. (2005). Transcriptional coactivator PGC-1 alpha controls the energy state and contractile function of cardiac muscle. Cell Metab..

[59] Kaur S., Bhatti G.K., Khullar N., Bhatti J.S. (2025). Targeting Mitochondrial microRNAs in Cardiovascular Pathologies: A New Frontier in Precision Cardiology. Ageing Res. Rev..

[60] Ha M., Kim V.N. (2014). Regulation of microRNA biogenesis. Nat. Rev. Mol. Cell Biol..

[61] Derda A.A., Pfanne A., Bär C., Schimmel K., Kennel P.J., Xiao K., Schulze P.C., Bauersachs J., Thum T. (2018). Blood-based microRNA profiling in patients with cardiac amyloidosis. PLoS ONE.

[62] Vita G.L., Aguennouz M., Polito F., Oteri R., Russo M., Gentile L., Barbagallo C., Ragusa M., Rodolico C., Di Giorgio R.M. (2020). Circulating microRNAs Profile in Patients with Transthyretin Variant Amyloidosis. Front. Mol. Neurosci..

[63] Mendell J.T., Olson E.N. (2012). MicroRNAs in stress signaling and human disease. Cell.

[64] Small E.M., Olson E.N. (2011). Pervasive roles of microRNAs in cardiovascular biology. Nature.

[65] Falk R.H. (2005). Diagnosis and management of the cardiac amyloidoses. Circulation.

[66] Patel K.S., Hawkins P.N. (2015). Cardiac amyloidosis: Where are we today?. J. Intern. Med..

[67] Thum T., Gross C., Fiedler J., Fischer T., Kissler S., Bussen M., Galuppo P., Just S., Rottbauer W., Frantz S. (2008). MicroRNA-21 contributes to myocardial disease by stimulating MAP kinase signalling in fibroblasts. Nature.

[68] Tijsen A.J., Pinto Y.M., Creemers E.E. (2012). Non-cardiomyocyte microRNAs in heart failure. Cardiovasc. Res..

[69] O’Connell R.M., Rao D.S., Baltimore D. (2012). microRNA regulation of inflammatory responses. Annu. Rev. Immunol..

[70] Montgomery R.L., Yu G., Latimer P.A., Stack C., Robinson K., Dalby C.M., Kaminski N., van Rooij E. (2014). MicroRNA mimicry blocks pulmonary fibrosis. EMBO Mol. Med..

[71] Boon R.A., Iekushi K., Lechner S., Seeger T., Fischer A., Heydt S., Kaluza D., Tréguer K., Carmona G., Bonauer A. (2013). MicroRNA-34a regulates cardiac ageing and function. Nature.

[72] Tian Z.Q., Jiang H., Lu Z.B. (2018). MiR-320 regulates cardiomyocyte apoptosis induced by ischemia-reperfusion injury by targeting AKIP1. Cell Mol. Biol. Lett..

[73] Yuan L., Fan L., Li Q., Cui W., Wang X., Zhang Z. (2019). Inhibition of miR-181b-5p protects cardiomyocytes against ischemia/reperfusion injury by targeting AKT3 and PI3KR3. J. Cell Biochem..

[74] Taganov K.D., Boldin M.P., Chang K.J., Baltimore D. (2006). NF-kappaB-dependent induction of microRNA miR-146, an inhibitor targeted to signaling proteins of innate immune responses. Proc. Natl. Acad. Sci. USA.

[75] Wang S., Aurora A.B., Johnson B.A., Qi X., McAnally J., Hill J.A., Richardson J.A., Bassel-Duby R., Olson E.N. (2008). The endothelial-specific microRNA miR-126 governs vascular integrity and angiogenesis. Dev. Cell.

[76] Merlini G., Palladini G. (2013). Light chain amyloidosis: The heart of the problem. Haematologica.

[77] Maurer M.S., Schwartz J.H., Gundapaneni B., Elliott P.M., Merlini G., Waddington-Cruz M., Kristen A.V., Grogan M., Witteles R., Damy T. (2018). Tafamidis Treatment for Patients with Transthyretin Amyloid Cardiomyopathy. N. Engl. J. Med..

[78] Etheridge A., Lee I., Hood L., Galas D., Wang K. (2011). Extracellular microRNA: A new source of biomarkers. Mutat. Res..

[79] Kontaraki J.E., Marketou M.E., Zacharis E.A., Parthenakis F.I., Vardas P.E. (2014). Differential expression of vascular smooth muscle-modulating microRNAs in human peripheral blood mononuclear cells: Novel targets in essential hypertension. J. Hum. Hypertens..

[80] Marketou M., Kontaraki J., Papadakis J., Kochiadakis G., Vrentzos G., Maragkoudakis S., Fragkiadakis K., Katsouli E., Plataki M., Patrianakos A. (2019). Platelet microRNAs in hypertensive patients with and without cardiovascular disease. J. Hum. Hypertens..

[81] Weng L., Spencer B.H., SoohHoo P.T., Connors L.H., O’Hara C.J., Seldin D.C. (2011). Dysregulation of miRNAs in AL amyloidosis. Amyloid.

[82] Marketou M., Kontaraki J., Patrianakos A., Kochiadakis G., Anastasiou I., Fragkiadakis K., Plevritaki A., Papadaki S.T., Chlouverakis G., Parthenakis F. (2021). Peripheral Blood MicroRNAs as Potential Biomarkers of Myocardial Damage in Acute Viral Myocarditis. Genes.

[83] Castiglione V., Franzini M., Aimo A., Carecci A., Lombardi C.M., Passino C., Rapezzi C., Emdin M., Vergaro G. (2021). Use of Biomarkers to Diagnose and Manage Cardiac Amyloidosis. Eur. J. Heart Fail..

[84] Palladini G., Dispenzieri A., Gertz M.A., Kumar S., Wechalekar A., Hawkins P.N., Schönland S., Hegenbart U., Comenzo R., Kastritis E. (2012). New criteria for response to treatment in immunoglobulin light chain amyloidosis based on free light chain measurement and cardiac biomarkers: Impact on survival outcomes. J. Clin. Oncol..

[85] Patrick D.M., Montgomery R.L., Qi X., Obad S., Kauppinen S., Hill J.A., van Rooij E., Olson E.N. (2010). Stress-dependent cardiac remodeling occurs in the absence of microRNA-21 in mice. J. Clin. Investig..

[86] Rupaimoole R., Slack F.J. (2017). MicroRNA therapeutics: Towards a new era for the management of cancer and other diseases. Nat. Rev. Drug Discov..

[87] Adams D., Gonzalez-Duarte A., O’Riordan W.D., Yang C.C., Ueda M., Kristen A.V., Tournev I., Schmidt H.H., Coelho T., Berk J.L. (2018). Patisiran, an RNAi Therapeutic, for Hereditary Transthyretin Amyloidosis. N. Engl. J. Med..

[88] Klimczak-Tomaniak D., Haponiuk-Skwarlińska J., Kuch M., Pączek L. (2022). Crosstalk between microRNA and Oxidative Stress in Heart Failure: A Systematic Review. Int. J. Mol. Sci..

[89] Gilyazova I., Timasheva Y., Chumakova A., Abdeeva G., Plotnikova M., Zagidullin N. (2025). The Role of MicroRNAs in the Pathophysiology and Management of Heart Failure: From Molecular Mechanisms to Clinical Application. Int. J. Mol. Sci..

[90] Perfetto F., Zampieri M., Fumagalli C., Allinovi M., Cappelli F. (2022). Circulating biomarkers in diagnosis and management of cardiac amyloidosis: A review for internist. Intern. Emerg. Med..

[91] Spahillari A., Jackson L., Varrias D., Michelhaugh S.A., Januzzi J.L., Shahideh B., Daghfal D., Valkov N., Murtagh G., Das S. (2024). MicroRNAs are Associated with Cardiac Biomarkers, Cardiac Structure and Function and Incident Outcomes in Heart Failure. ESC Heart Fail..

[92] Galluzzo A., Gallo S., Pardini B., Birolo G., Fariselli P., Boretto P., Vitacolonna A., Peraldo-Neia C., Spilinga M., Volpe A. (2021). Identification of Novel Circulating microRNAs in Advanced Heart Failure by Next-Generation Sequencing. ESC Heart Fail..

[93] McDonald J.S., Milosevic D., Reddi H.V., Grebe S.K., Algeciras-Schimnich A. (2011). Analysis of circulating microRNA: Preanalytical and analytical challenges. Clin. Chem..

